# Maternal Deaths Due to Suicide, Accidental Poisoning and Undetermined Intent Within 5 Years Following Childbirth: A Population‐Based Study

**DOI:** 10.1111/1471-0528.70212

**Published:** 2026-03-31

**Authors:** Louise Makarious, Ju Lee Oei, Andrew Page, Sadia Hossain, Mithilesh Dronavalli, Hannah Uebel, Evelyn Lee, Michelle Dickson, Lucinda Burns, Barbara Bajuk, John Eastwood, Sithum Munasinghe

**Affiliations:** ^1^ Department of Obstetrics and Gynaecology Westmead Hospital Westmead New South Wales Australia; ^2^ Discipline of Paediatrics and Child Health, Faculty of Medicine and Health, School of Clinical Medicine University of New South Wales Randwick New South Wales Australia; ^3^ Department of Newborn Care Royal Hospital for Women Randwick New South Wales Australia; ^4^ Translational Health Research Institute Western Sydney University Penrith New South Wales Australia; ^5^ School of Health Sciences Western Sydney University Campbelltown New South Wales Australia; ^6^ Department of Paediatrics Sydney Children's Hospital Sydney New South Wales Australia; ^7^ Leeder Centre for Health Policy, Economics & Data, Faculty of Medicine and Health University of Sydney Sydney New South Wales Australia; ^8^ The Poche Centre for Indigenous Health, Faculty of Medicine and Health University of Sydney Camperdown New South Wales Australia; ^9^ National Drug and Alcohol Research Centre University of New South Wales Randwick New South Wales Australia; ^10^ Critical Care Program Sydney Children's Hospitals Network Sydney New South Wales Australia; ^11^ National Public Health Service Te Whatu Ora—Health New Zealand Dunedin New Zealand; ^12^ School of Population Health University of New South Wales Randwick New South Wales Australia

**Keywords:** accidental poisoning, deaths due to undetermined intent, maternal mortality, postpartum, suicide

## Abstract

**Objectives:**

Investigate the incidence of maternal deaths by suicide, accidental poisoning and undetermined intent within 5 years following childbirth.

**Design:**

Linked population‐level data.

**Setting:**

New South Wales, Australia.

**Population:**

All women who gave birth from 2002 to 2020.

**Methods:**

Sociodemographic characteristics, diagnostic and healthcare use were compared between deceased mothers and mothers alive for 5 years following childbirth. Relative Risk Ratios (RRRs) were examined using multinomial logistic regression.

**Main Outcome Measures:**

Deaths by suicide, undetermined intent and accidental poisoning.

**Results:**

Five‐year death rates per 100 000 live births were 12.87 for suicide, 10.49 for accidental poisoning, 2.30 for undetermined intent and 25.66 for the three causes and remained stable over the study period. Deaths due to the abovementioned causes accounted for 21.75% of all deaths within 5 years after live birth, slightly increased as a proportion of all deaths compared to the early period, between 2002 and 2009. Compared to mothers who were alive 5 years postpartum, mothers who died by suicide, accidental poisoning and undetermined intent were more likely to be younger (age ≤ 24) (25% vs. 9%), Australian born (80+% vs. 65%), and identify as First Nations background (12+% vs. 2.3%). The RRRs were significantly higher for alcohol (ranged 15–27), substance use (ranged 12–69), mental health (ranged 9.5–14) and self‐harm (ranged 20–34), related hospitalisations among mothers who died by suicide, accidental poisoning and undetermined intent compared to mothers alive for 5 years.

**Conclusion:**

Suicide, undetermined intent and accidental poisoning accounted for more than one in every five maternal deaths within 5 years postpartum.

## 1 Introduction

Suicide remains a significant health burden globally, yet one of the most preventable causes of death [1]. Motherhood is associated with lower rates of suicide compared to women overall [2], but one of the leading causes of death during the postpartum period, with previous research indicating that between 2 and 4 mothers die by suicide per 100 000 live births in the first year after childbirth [3, 4, 5, 6, 7]. However, in Australia, suicide ranked as the fourth leading cause of mortality during pregnancy and 42 days after the end of pregnancy [8], and the second leading cause of mortality within the first year after pregnancy [9]. Despite years of research and targeted improvements in clinical practice to reduce maternal mortality due to haemorrhage, blood pressure disorders and infections, non‐obstetric causes (e.g., mental health and psychosocial conditions), have become the leading causes of mortality, with suicide accounting for between 13% and 36% of all maternal deaths in the first year postpartum [10].

Although substantial research has been conducted on suicide in the postpartum period, there are inconsistencies in the enumeration of suicides. Some studies considered events due to undetermined intent as suicides [2, 6, 11], while others only enumerated certain suicides [5, 12]. Previous research suggests that deaths from suicide may be underreported due to misclassification as deaths caused by unintentional poisoning or undetermined intent [13, 14]. A recent review showed that deaths from suicide and accidental poisoning share common risk factors, including psychiatric conditions and suicidal behaviour [15]. However, only a small number of studies have considered maternal deaths from suicide and accidental poisoning, predominantly based in the US [16, 17, 18, 19].

The early period after childbirth is a higher‐risk period for suicidal behaviour for mothers, given that mothers are more likely to experience postnatal depression and other mental health conditions [2, 20, 21]. Previous research examining maternal suicide was mainly designed to investigate mothers who die within 1 year following childbirth, whilst no studies extended the period beyond 1 year. The mother–child relationship is critical to a child's cognitive and social development [22], and losing a mother in childhood can have lasting effects [23].

Accordingly, this study was designed to examine the incidence of maternal deaths by suicide, accidental poisoning and undetermined intent within 5 years following childbirth. Additionally, this study investigates sociodemographic characteristics, diagnostic profile and previous health service use of mothers who died by suicide, accidental poisoning and undetermined intent, compared with mothers who died from other causes and mothers who were alive up to 5 years following childbirth in NSW, Australia.

## 2 Methods

### 2.1 Data Sources

This study used six data sources, including the NSW Perinatal Data Collection (PDC), with data availability from July 2001 to December 2020. The PDC was linked to the NSW Registry for Births, Deaths and Marriages (RBDM), the NSW Cause of Death Record File (CDRF), the NSW Mental Health Ambulatory Care Data Collection (MHACDC), NSW Family and Community Services—ChildStory (KiDS migrated to ChildStory in 2017) data collection (DCJ ChildStory; Out of Home Care (OOHC) placement information), and the NSW Admitted Patient Data Collection (APDC). The data linkage was conducted by the NSW Centre for Health Record Linkage (CheReL) using probabilistic record linkage techniques, and a detailed description explaining each data source and data linkage has been published elsewhere [24]. Briefly, the PDC contains information on the mother's sociodemographic characteristics, pregnancy‐related health conditions, antenatal care history, type of delivery, birth complications and information related to the baby's health and healthcare utilisation before discharge from the hospital following delivery.

The RBDM contains information related to birth date and date of death, whereas the CDRF collected the cause of death recorded using the International Statistical Classification of Diseases, tenth revision (ICD‐10) codes [25]. The APDC collects information related to hospital admissions, diagnoses (coded in ICD‐10 Australian Modification) and discharge summaries. The DCJ ChildStory OOHC captures care start and end dates, priority placement purpose and type, care type and type of caregiver. The MHACDC comprises information related to mental health diagnoses, information on psychological interventions and session‐ and activity‐related characteristics.

### 2.2 Study Design and Procedure

This retrospective longitudinal birth cohort study was based in New South Wales (NSW), Australia, and used routinely collected retrospective data to enumerate all live births and corresponding information on the cohort of mothers who gave live birth. The current study included all mothers who gave live birth between January 2002 and December 2020. During the study period, 1 006 592 mothers gave birth to 1 780 258 infants. Since every dataset starts in July 2001, including the PDC, we have shifted the cohort start date from January 2002 to allow a 6‐month look‐back period to investigate previous healthcare utilisation for mothers who gave live birth.

### 2.3 Study Variables

The outcome of this study was maternal death within 5 years following childbirth due to suicide, accidental poisoning, or undetermined intent. The cause of death was identified based on the ICD‐10 codes recorded in the primary cause of death field and classified as suicide (ICD‐10 codes X60‐84), deaths due to accidental poisoning (X40‐49), and deaths due to undetermined intent (Y10‐34). This study also presented deaths from other causes within 5 years following childbirth to compare study characteristics (see below) between mothers who died by suicide, accidental poisoning and undetermined intent.

The study outcome was stratified by a range of study variables, including socio‐demographic information, pregnancy‐related characteristics, child placement in OOHC services and mother's previous hospital presentations related to drug use, mental health conditions, suicidal behaviour, poisoning and other undetermined intent and mental health care service utilisation. Classification of hospital presentations related to the above conditions was based on the primary diagnosis field and 50 additional diagnosis fields in the APDC (ICD‐10 codes used to identify each diagnosis are summarised in Table S1).

Socio‐demographic details included maternal age at childbirth (‘≤ 24’, ‘25–29’, ‘30–34’, ‘≥ 35’), country of birth (‘Australia’, ‘others’), First Nations status, classified as ‘Yes’ for mothers with Aboriginal and Torres Strait Islander background, previous pregnancies (‘No’, ‘Yes’), area‐level socioeconomic status measured in terms of the Index of Relative Socioeconomic Disadvantages (IRSD) [26] and the Accessibility and Remoteness Index of Australia (ARIA) to define urban–rural residence [27]. Pregnancy‐related characteristics included the number of weeks until the first antenatal care visit and the number of antenatal care visits.

Drug‐related hospitalisation included any hospital presentation related to alcohol, opioids, cannabinoids and other presentations due to drug abuse (Table S1). Hospital‐presenting mental health conditions were grouped as any presentation related to affective disorders, anxiety disorders and other mental health disorders, except drug use. Additionally, any hospital presentations due to self‐harm, suicidal ideation, events due to undetermined intent, presentations due to accidental poisoning, and the number of visits to mental health ambulatory care services 12 months before death (‘0’, ‘1’, ‘2–3’, ‘4–6’, ‘≥ 7’).

### 2.4 Data Analysis

Descriptive analyses were conducted to summarise data, including graphs and tables. Trends in maternal deaths were presented as (i) rates per 100 000 live births and (ii) as a proportion of all deaths, within 1, 2 and 5 years following a live birth, by each calendar year using graphs. To calculate maternal deaths (as a rate per 100 000 live births and as a percentage of all deaths) within 5 years after a live birth, the cohort period was restricted from January 2002 to December 2015, allowing for a 5‐year follow‐up period. Similarly, for maternal deaths within 1 and 2 years, the cohort period was set to January 2002 to December 2019 and January 2002 to December 2018, respectively.

Additionally, this study compared study characteristics between mothers who died by suicide, accidental poisoning, and undetermined intent, with mothers who died from other causes and mothers who were alive for 5 years. For this comparison, the cohort for mothers alive for 5 years was restricted to the period from January 2002 to December 2015, as we have follow‐up data until December 2020. However, the full cohort period from January 2002 to December 2020 was considered for mothers who died within 5 years following a live birth. Because some mothers had multiple births at different times, the birth date of the last live birth record was considered to identify whether the mother died within 1, 2, or 5 years in the mother‐level analyses. Counts and percentages were used to summarise categorical variables, whereas median and interquartile range (IQR) were used to summarise continuous variables. Cell counts and marginal totals in tables were suppressed if the cell counts were < 5. Multinomial univariate logistic regression models were employed to obtain relative risk ratios for suicide, deaths due to accidental poisoning and undetermined intent, compared with the cohort of live mothers and mothers who died within 5 years due to other causes. Negative binomial models were also employed to estimate the association between study factors and maternal mortality in the presence of rare events [28]. In the univariate analysis, Opioids and cannabinoid drug types were combined, and mental health condition types were also combined to avoid large confidence intervals. All the analyses were conducted using Stata version 17.0 (Stata Corp, College Station, Texas, USA), and R Studio version 4.2.

## 3 Results

From 2002 to 2020 in NSW, Australia, there were 161 deaths due to suicide, 126 deaths due to accidental poisoning and 27 deaths due to undetermined intent within 5 years following the live birth.

### 3.1 Death Rates and Percentages Within 1 and 5 Years Following Childbirth

**FIGURE 1:**
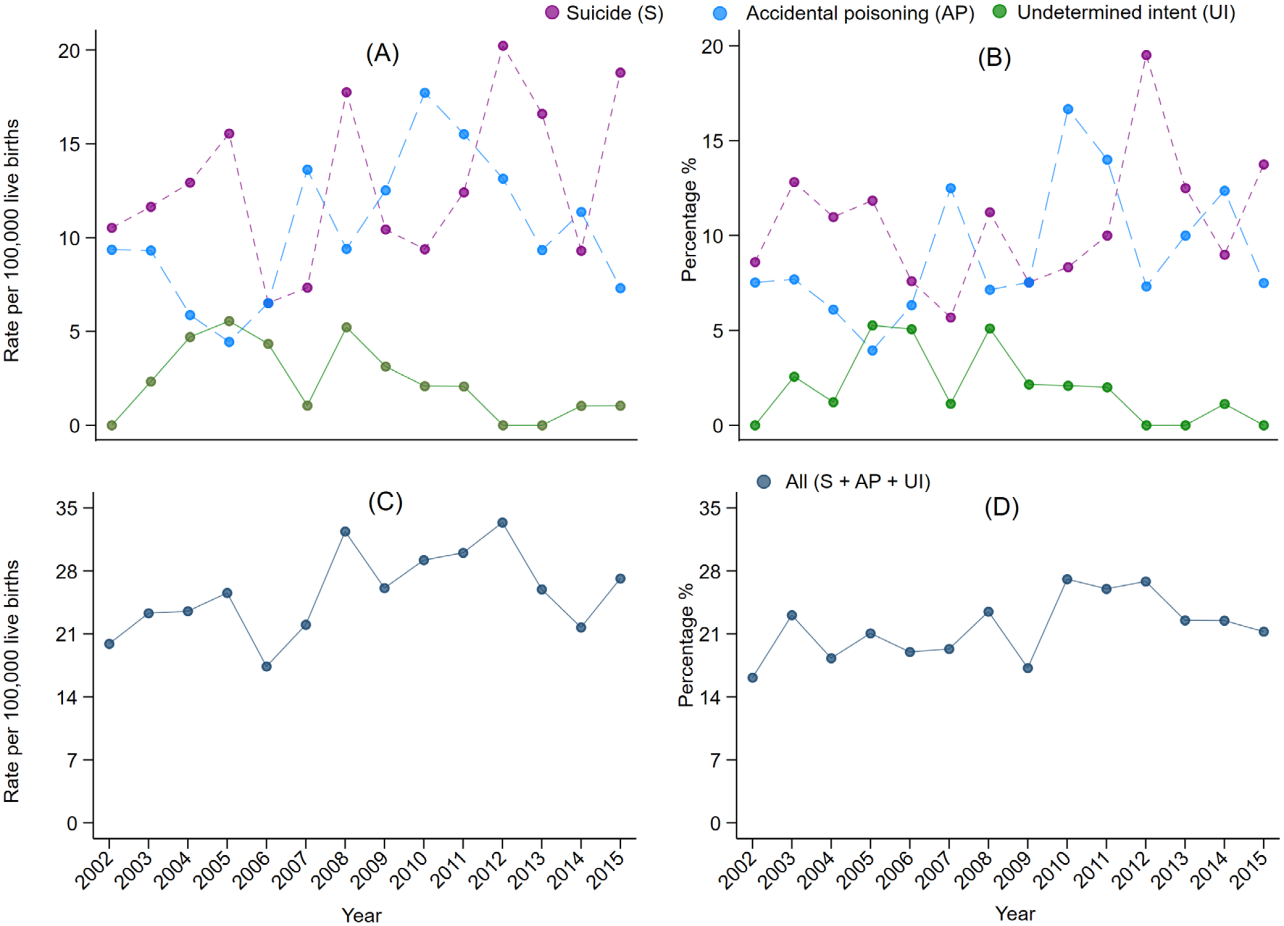
Deaths due to suicide, accidental poisoning and undetermined intent within 5 years following a live birth in NSW from 2002 to 2020. (Panel A) The rate of 5‐year maternal mortality by suicide, accidental poisoning and undetermined intent per 100 000 live births; (Panel B) Maternal mortality by suicide, accidental poisoning and undetermined intent as a percentage of all deaths within 5 years following a live birth; (Panel C) The rate of combined 5‐year maternal mortality by suicide, accidental poisoning and undetermined intent per 100 000 live births; (Panel D) Combined maternal mortality by suicide, accidental poisoning and undetermined intent as a percentage of all deaths within 5 years following a live birth. The period after 2015 was not considered due to the unavailability of a 5‐year follow‐up period. Since a mother can give birth to multiple children at different times within 5 years, the rates in Panels A and C are birth‐specific and capture all live births, and the rate is presented per 100 000 live births. For panels B and D, the birth date of the last live birth was considered to calculate time to death if mothers gave multiple childbirths at different time points.

Mortality rates per 100 000 live births were 12.9 for suicide, 10.5 for accidental poisoning and 2.3 for undetermined intent over the first 5 years and remained relatively stable throughout the study period (Figure 1 and Figure S3). The 12‐month death rates were 2.8, 1.1 and 0.5 for suicide, accidental poisoning and undetermined intent, respectively (Figure S3). Deaths from suicide, accidental poisoning and undetermined intent represented 26% of all deaths in the first year, 23% of all deaths in the first 2 years, and 22% of all deaths in the first 5 years following childbirth, and the deaths related to these three causes as a proportion of all deaths slightly increased over the study period (Figure 1, Figures S1–S3). Suicide (25.7%) and deaths from undetermined intent (33.3%) were more likely to occur in the first year in comparison to deaths from accidental poisoning (14.3%) within the 5 years. However, deaths by suicide, accidental poisoning and undetermined intent combined remained relatively stable in each year over the 5 years following childbirth (Figure S4).

### 3.2 Demographic Profiles and Antenatal Care

Compared to mothers who were alive 5 years post‐partum, mothers who died by suicide, accidental poisoning and undetermined intent combined were more likely to be younger (≤ 24 years) (25% vs. 9%), born in Australia (80+% vs. 65%), nulliparous (30+% vs. 25%), identify as First Nations background (12+% vs. 2.3%), and live in the remote (28% vs. 21%) and two most socio‐economically disadvantaged population quintiles (55% vs. 43%) (Table 1). Nearly 50% of mothers who died by accidental poisoning or undetermined intent had their children placed in OOHC services at some point prior to death (Table 1). In contrast, the previous figure was 12% for mothers who died by suicide, 6.8% for mothers who died from other causes and 1.3% for mothers who were alive for at least 5 years, respectively (Table 1). Compared to mothers who died by suicide (median = 10 weeks; IQR = 6 weeks, 18 weeks), mothers who died by accidental poisoning (median = 14 weeks; IQR = 8 weeks, 20 weeks) and undetermined intent (median = 12 weeks; IQR = 7 weeks, 18 weeks) accessed antenatal care visits later. Further, mothers who died from accidental poisoning (median = 8; IQR = 5, 10) and undetermined intent (median = 9.5, IQR = 5.5, 11.5) had lower total antenatal care visits compared to mothers who died by suicide (median = 10, IQR = 8, 11) (Table 1).

**TABLE 1:** Socio‐demographic characteristics of women in NSW who died from 2002 to 2020 by suicide, accidental poisoning and due to undetermined intent within 5 years after childbirth.

Characteristics	Suicide (*n* = 161)	Accidental poisoning (*n* = 126)	Events due to undetermined intent (*n* = 27)	All three causes (*n* = 314)	all deaths (other causes) (*n* = 1094)	Alive (*n* = 625 856)
**Age at last childbirth**						
≤ 24	47 (29.19)	25 (19.84)	8 (29.63)	80 (25.48)	144 (13.16)	54 320 (8.68)
25–29	48 (29.81)	35 (27.78)	6 (22.22)	89 (28.34)	205 (18.74)	127 828 (20.42)
30–34	36 (22.36)	40 (31.75)	7 (25.93)	83 (26.43)	310 (28.34)	226 005 (36.11)
≥ 35	30 (18.63)	26 (20.63)	6 (22.22)	62 (19.75)	435 (39.76)	217 703 (34.78)
**Age at first childbirth** ^**a**^						
≤ 24	54 (48.21)	39 (53.42)	6 (54.55)	99 (50.51)	189 (28.21)	84 671 (20.89)
≥ 25	58 (51.79)	34 (46.58)	5 (45.45)	97 (49.49)	481 (71.79)	320 551 (79.1)
Age at first childbirth not recorded^b^	49	53	16	118	424	220 634
**First nations status**						
No	148 (91.93)	102 (80.95)	np	np	1000 (91.49)	610 531 (97.69)
Yes	13 (8.07)	24 (19.05)	np	np	93 (8.51)	14 448 (2.31)
**Country of birth**						
Australia	123 (76.4)	114 (90.48)	np	np	801 (73.42)	407 672 (65.31)
Other	38 (23.6)	12 (9.52)	np	np	290 (26.58)	216 491 (34.69)
**Remoteness**						
Major cities	116 (72.5)	93 (75)	15 (55.56)	224 (72.03)	766 (70.93)	484 664 (78.67)
Regional/remote	44 (27.5)	31 (25)	12 (44.44)	87 (27.97)	314 (29.07)	131 432 (21.33)
**IRSD**						
1 Most deprived	29 (18.01)	32 (25.81)	8 (29.63)	69 (22.12)	260 (23.92)	124 100 (20.14)
2	54 (33.54)	39 (31.45)	9 (33.33)	102 (32.69)	309 (28.43)	143 141 (23.23)
3	36 (22.36)	29 (23.39)	5 (18.52)	70 (22.44)	202 (18.58)	119 133 (19.34)
4	23 (14.29)	11 (8.87)	5 (18.52)	39 (12.5)	164 (15.09)	100 700 (16.34)
5 Least deprived	19 (11.8)	13 (10.48)	0 (0)	32 (10.26)	152 (13.98)	129 022 (20.94)
**Previous pregnancies**						
No	57 (35.4)	36 (28.57)	np	np	319 (29.19)	153 628 (24.56)
One	55 (34.16)	29 (23.02)	np	np	346 (31.66)	265 903 (42.51)
Two or more	49 (30.43)	61 (48.41)	12 (44.44)	122 (38.85)	428 (39.16)	205 979 (32.93)
**First antenatal care visit** ^**c**^						
Weeks of pregnancy	10 (6,18)	14 (8,20)	12 (7,18)	12 (7,18)	11 (7,16)	10 (7,15)
Number of antenatal care visits	10 (8,11)	8 (5,10)	9.5 (5.5,11.5)	10 (6,10)	10 (8,11)	10 (8,11)
**Child placed in OOHC before mother dies** ^**d**^						
No	141 (87.58)	64 (50.79)	14 (51.85)	219 (69.75)	1020 (93.24)	617 573 (98.67)
Yes	20 (12.42)	62 (49.21)	13 (48.15)	95 (30.25)	74 (6.76)	8304 (1.33)

*Note:* np‐ cell counts and percentages were not provided (np) due to low cell counts.

^a^ Age of mothers when their first live birth occurred, and the records were excluded if the mother's age was not available for their first live child's birth, only when calculating this statistic.

^b^ Only counts were presented without percentages; IRSD‐Index of relative socioeconomic disadvantage.

^c^ Median and interquartile range were presented.

^d^ Atleast one child (if mothers have multiple children) was placed in OOHC at some point of time prior to the mother's death, but this doesn't mean the child was in OOHC when the mother died.

### 3.3 Previous Hospital Presentations

**FIGURE 2:**
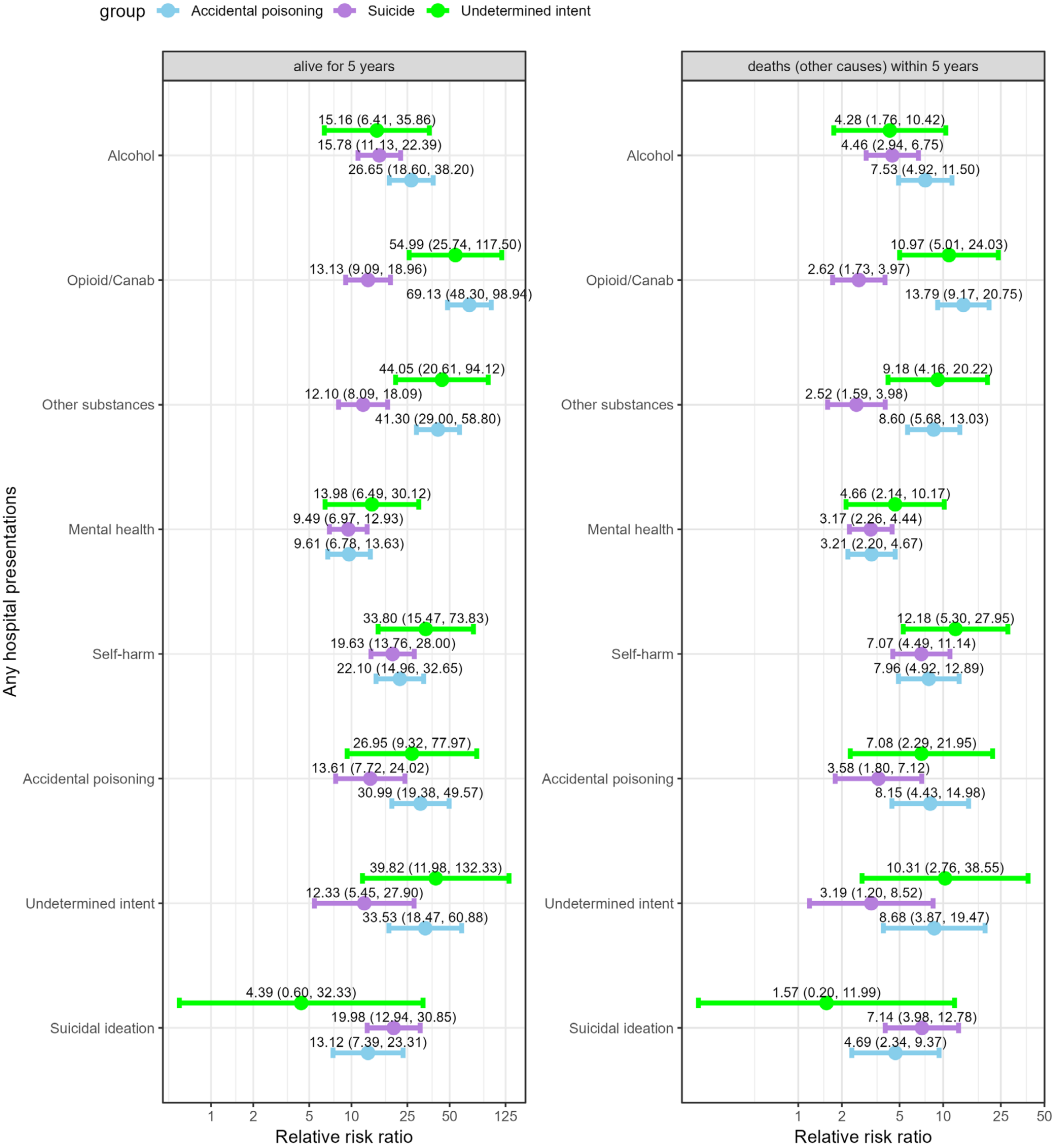
Relative risk ratios of hospital‐presented conditions among mothers who died by suicide, accidental poisoning and undetermined intent compared to mothers who were alive for 5 years and died by other causes within 5 years. Nicotine, Cocaine and Hallucinogens were combined with other substances due to the low frequencies. The reference of the first panel is mothers who were alive for 5 years, whereas the reference of the second panel is deaths due to other causes within 5 years. Univariate multinomial logistic regression models were employed to obtain Relative risk ratios.

Nearly one‐third of mothers who died by suicide, accidental poisoning and undetermined intent combined had presented (before or after pregnancy) to the hospital related to anxiety (31.2%), affective (34.4%) and alcohol use disorders (31.2%). Compared to mothers who died by suicide, mothers who died from accidental poisoning and undetermined intent had higher hospital‐presented episodes for opioids (50% and 40.7% vs. 8.7%), cannabinoids (31.8% and 29.6% vs. 17.4%), alcohol (38.1% and 25.9% vs. 26.7%) and other substance (42.9% and 44.4% vs. 18%) use disorders (Table 2). In contrast, nearly 6%–8% of mothers who died from other causes and 1%–2.3% of mothers alive up to 5 years postpartum were presented to the hospital related to drug and alcohol disorders (Table 2). Compared to mothers alive for 5 years following childbirth, mothers who died by suicide, accidental poisoning and undetermined intent within 5 years had a higher likelihood of any hospital presentations related to alcohol (RRRs ranged from 15.2 to 26.7; 95% CI ranged from 6.4 to 38.2), Opioid/Cannabinoid (RRRs ranged from 13.1 to 69.1; 95% CI ranged from 9.1 to 117.5), other substances (RRRs ranged from 12.1 to 44.1; 95% CI ranged from 8.1 to 94.1), any mental health (RRRs ranged from 9.5 to 14; 95% CI ranged from 6.5 to 30.1) and self‐harm (RRRs ranged from 19.6 to 33.8; 95% CI ranged from 13.8 to 73.8) (Figure 2, Table S2). Similarly, the RRRs of the above risk factors were significantly higher for mothers who died from suicide, accidental poisoning and undetermined intent compared to mothers who died from other causes (Figure 2, Table S2).

**TABLE 2:** Causes of hospital presentations in women who died by suicide, accidental poisoning and due to undetermined intent within 5 years following childbirth in NSW from 2002 to 2020.

Admission reason	Suicide (*n* = 161)	Accidental poisoning (*n* = 126)	Events due to undetermined intent (*n* = 27)	All three causes (*n* = 314)	All deaths (other causes) (*n* = 1094)	Alive (*n* = 625 856)
**Alcohol**						
No	118 (73.29)	78 (61.9)	20 (74.07)	216 (68.79)	1003 (92.44)	610 259 (97.74)
Yes	43 (26.71)	48 (38.1)	7 (25.93)	98 (31.21)	82 (7.56)	14 090 (2.26)
**Opioids**						
No	147 (91.3)	63 (50)	16 (59.26)	226 (71.97)	1014 (93.46)	617 585 (98.92)
Yes	14 (8.7)	63 (50)	11 (40.74)	88 (28.03)	71 (6.54)	6764 (1.08)
**Cannabinoids**						
No	133 (82.61)	86 (68.25)	19 (70.37)	238 (75.8)	1023 (94.29)	615 083 (98.52)
Yes	28 (17.39)	40 (31.75)	8 (29.63)	76 (24.2)	62 (5.71)	9266 (1.48)
**Affective disorders**						
No	102 (63.35)	88 (69.84)	16 (59.26)	206 (65.61)	956 (88.11)	598 757 (95.9)
Yes	59 (36.65)	38 (30.16)	11 (40.74)	108 (34.39)	129 (11.89)	25 592 (4.1)
**Anxiety disorders**						
No	112 (69.57)	88 (69.84)	16 (59.26)	216 (68.79)	926 (85.35)	583 894 (93.52)
Yes	49 (30.43)	38 (30.16)	11 (40.74)	98 (31.21)	159 (14.65)	40 455 (6.48)
**Other MH disorders**						
No	120 (74.53)	89 (70.63)	18 (66.67)	227 (72.29)	976 (89.95)	604 422 (96.81)
Yes	41 (25.47)	37 (29.37)	9 (33.33)	87 (27.71)	109 (10.05)	19 927 (3.19)
**Other substance use disorders**						
No	132 (81.99)	72 (57.14)	15 (55.56)	219 (69.75)	998 (91.98)	613 212 (98.22)
Yes	29 (18.01)	54 (42.86)	12 (44.44)	95 (30.25)	87 (8.02)	11 137 (1.78)
**Self‐harm**						
No	120 (74.53)	91 (72.22)	17 (62.96)	228 (72.61)	1035 (95.39)	613 669 (98.29)
Yes	41 (25.47)	35 (27.78)	10 (37.04)	86 (27.39)	50 (4.61)	10 680 (1.71)
**Accidental poisoning**						
No	148 (91.93)	105 (83.33)	np	np	1059 (97.6)	620 346 (99.36)
Yes	13 (8.07)	21 (16.67)	np	np	26 (2.4)	4003 (0.64)
**Events due to undetermined intent**						
No	155 (96.27)	114 (90.48)	np	np	1072 (98.8)	622 395 (99.69)
Yes	6 (3.73)	12 (9.52)	np	np	13 (1.2)	1954 (0.31)
**Suicidal ideation**						
No	137 (85.09)	113 (89.68)	np	np	1059 (97.6)	618 922 (99.13)
Yes	24 (14.91)	13 (10.32)	np	np	26 (2.4)	5427 (0.87)
**Mental health service visits last 12 months** ^**a**^						
No	95 (59.01)	83 (65.87)	18 (66.67)	196 (62.42)	978 (89.4)	NA
Yes	66 (40.99)	43 (34.13)	9 (33.33)	118 (37.58)	116 (10.6)	NA

*Note:* Hospital presentations included any presentations to the hospital before or after the live birth during the study period. Hospital presentations of patients who were discharged on or before the day of death were excluded to ensure that the diagnosis was not based on the episode of death. np‐ cell counts and percentages were not provided (np) due to low cell counts. Nicotine, Cocaine and Hallucinogens were combined with other substances due to the low frequencies.

Abbreviation: NA, not applicable.

^a^ Mental health service visits last 12 months indicate any outpatient mental health service attendance.

### 3.4 Mental Health Service Utilisation

Nearly 60% of mothers who died by suicide, and two‐thirds of mothers who died from accidental poisoning (65.9%) and undetermined intent (66.7%) did not visit community mental health services within 12 months before death (Table 2). The frequency of visits to community mental health services increased within the last 3 months before death among mothers who died by suicide, whereas it reduced for mothers who died by accidental poisoning and remained unchanged for mothers who died by undetermined intent (Figure S5).

## 4 Discussion

### 4.1 Main Findings

This study examined the incidence of maternal deaths by suicide, accidental poisoning and undetermined intent within 5 years following childbirth, and investigated sociodemographic characteristics, diagnoses and previous health services use for mothers who died from the above three causes, compared to mothers who died from other causes and mothers who were alive for 5 years. Findings suggest that more than one in every five deaths were attributable to suicide, undetermined intent and accidental poisoning combined, with a slightly increasing trend over the study period as a percentage of all deaths compared to the early period. However, per 100 000 live births, deaths from these three causes remained relatively stable, with approximately 4.4 deaths within the first year and 25.7 deaths within 5 years. Findings also suggest that mothers who died by suicide, undetermined intent and accidental poisoning were more likely to be younger, from First Nations backgrounds, attended a lower number of antenatal care visits and more often had their children placed in OOHC before they died. Further, hospital presentations related to mental health conditions, drug abuse and suicidal behaviour were significantly higher among mothers who died by suicide, undetermined intent, and accidental poisoning compared to mothers who died from other causes or who were alive for 5 years following childbirth.

### 4.2 Interpretation

Current findings indicated 2.8 suicide deaths per 100 000 live births (3.3 when deaths due to undetermined intent were included), which was consistent with previous research [3, 6, 7], whereas 1.1 deaths due to accidental poisoning per 100 000 live births were lower compared to the US [18]. Rates per 100 000 live births remained relatively stable for suicide, undetermined intent and accidental poisoning combined, while a slight increase was observed as a proportion of all deaths over the recent period and this increment likely reflects the recent improvements in clinical practice that reduce maternal deaths due to infections and other obstetric complications [9]. Deaths due to suicide and undetermined intent were highest in the first year after live birth compared to accidental poisoning. The high risk of suicide among mothers within the first year may be partly associated with the transition to motherhood and parenting, negative self‐evaluation of caring for the child, and the responsibilities of motherhood [29].

Consistent with a wide body of literature, our findings indicated that mothers who died by suicide, undetermined intent, or accidental poisoning were younger at their first pregnancy, identified as First Nations, were more likely to reside in socioeconomically disadvantaged locations, and had previous presentations to hospitals related to mental disorders, drug abuse and suicidal behaviour [4, 9]. However, there were notable differences in certain risk factors leading to cause‐specific deaths. Compared to accidental poisoning deaths, mothers who died by suicide were more likely to be nulliparous and had presented to mental health services 12 months before they died. This is consistent with previous research that has shown nulliparity is more common among mothers who died by suicide compared to those who died by accidental poisoning [15], reflecting the specific challenges associated with first‐time motherhood, including motherhood responsibilities and negative self‐evaluation for caring for the child, highlighted by previous studies [29]. In contrast, First Nations background, alcohol and drug abuse and hospital presentation were more likely among mothers who died due to undetermined intent and accidental poisoning. Additionally, the time to attend the first antenatal care visit was longer, and the number of visits was lower among mothers who died by accidental poisoning. A study by Burns et al. [30] revealed that substance‐dependent women were less likely to attend antenatal care services. In Australia, there has been a recent implementation of a model of care [31] for mothers with substance use during pregnancy aimed to promote engagement in antenatal and postnatal care to provide ongoing community‐based support [31]. Further research is warranted to provide continuous service improvement to reduce substance‐related adverse health outcomes in this group. Furthermore, mothers who died by accidental poisoning and undetermined intent, compared to suicide, were more likely to have children placed in OOHC prior to death. This finding may indicate prior difficulties associated with alcohol and drug dependence and mental health to a degree that the child was deemed unsafe to live with the mother. It also indicates that there were opportunities during child protection interactions where the mothers could have been offered suitable health care and interventions.

Findings also demonstrated that nearly two‐thirds of mothers who died by suicide, undetermined intent, or accidental poisoning did not access ambulatory care mental health services. This finding is similar to previous research indicating that almost 60% of women diagnosed with perinatal depression did not engage in support despite referral [32], and two‐thirds of the general population who took their own life did not receive mental health treatments before suicide [33]. Mental health treatment non‐attendance [34] and disengagement [35] were shown to be associated with increased subsequent suicidal behaviour, and re‐engagement of those lost services was found to be the most effective intervention in reducing suicide and attempted suicide [36]. However, mental health treatments alone are not sufficient to reduce suicide deaths [37]. Prevention programs that address social determinants associated with suicide, such as financial problems, social isolation and relationship problems [38] and specific challenges associated with motherhood [29]. Additionally, service pathways for mothers with mental health problems with co‐morbid substance use disorders are recommended to reduce suicide and accidental poisoning deaths.

### 4.3 Limitations

There are several methodological limitations for consideration when interpreting findings of this study. First, deaths due to undetermined intent were unable to be classified as suicide or accidental poisoning due to ambiguity, as it is not possible to know with certainty the deceased person's intent and therefore may underestimate the rates of maternal mortality from suicide and accidental poisoning. Second, other well‐known risk factors for suicide, including intimate partner violence and relationship breakdown, and psychological stressors, were not included in this study [6, 39]. Third, the current study did not include Emergency Department data due to the unavailability of the presenting problem and SNOMED diagnostic description, and primary care presentations to general practice settings due to the unavailability of data. Therefore, the current study underestimates previous health service utilisation due to mental health, drug abuse, self‐harm and unintentional poisoning. Finally, the current study may underestimate the mothers who accessed mental health services 12 months before death because a group of them who did not access ambulatory care mental health services may have accessed other mental health services commissioned via Primary Health Networks [34], or other fee‐for‐service [40]. The generalisability of the study findings to other settings should take into account sociodemographic differences and variations in available resources.

Overall, our findings indicated that suicide, undetermined intent and accidental poisoning account for more than one in every five maternal deaths within 5 years postpartum. Recognition of at‐risk mothers and providing support beyond 1‐year postpartum is imperative to reduce preventable maternal death. Routine screening for risk factors for suicide and drug and alcohol abuse to facilitate treatments for mental health, drug and alcohol abuse is crucial for reducing deaths related to suicide, accidental poisoning and undetermined intent. Extending the period for screening and surveillance beyond 1 year by integrating obstetric and primary care services ensures continuity of care and timely monitoring for mothers at risk of suicidal behaviour should be a priority.

## Author Contributions

L.M., J.L.O., A.P., S.M. contributed to the study conception and design. J.L.O. sought ethics clearance and obtained access to linked data. L.M. wrote the first draft, and A.P. and S.M. critically revised the first draft. S.M. cleaned the data and conducted data analysis. All authors provided intellectual inputs on the first draft, contributed to interpreting data and revised the manuscript.

## Funding

All phases of the Joining the Dots Study were supported by the Mindgardens Neuroscience Network; Australian Red Cross; Alpha Maxx Healthcare; the Centre for Research Excellence for Integrated Health and Social Care (CREHSCI), funded by the National Health and Medical Research Council (grant number: APP1198477); and the University of Sydney.

## Ethics Statement

Access to the data of this study was in accordance with the ethics approvals by the NSW Population and Health Service (2019ETH12716) and the Australian Capital Territory Health (2021–1231, 2021–1232, 2021–1233) Human Research Ethics Committees, the Aboriginal Health and Medical Research Council (1824/21) and all the Australian education sectors: Board of Studies (government schools), Australian Independent Schools and Catholic Education Commission (D2014/120797).

## Consent

The authors have nothing to report.

## Conflicts of Interest

The authors declare no conflicts of interest.

## Supporting information


**Data S1:** bjo70212‐sup‐0001‐Supinfo.docx.

## Data Availability

Data for this study is not publicly available. Data accessibility requires ethics clearance and University of New South Wales affiliation.
