# Supplementary material for: Maternal Deaths Due to Suicide, Accidental Poisoning and Undetermined Intent Within 5 Years Following Childbirth: A Population‐Based Study
Source: BJOG. 2026 Mar 31;133(8):1581–91. doi: 10.1111/1471-0528.70212 (PMC13254012; doi:10.1111/1471-0528.70212)
Supplement: Supplementary file 1 — Data S1: bjo70212‐sup‐0001‐Supinfo.docx. [file BJO-133-1581-s001.docx]

**SUPPLEMENTARY MATERIAL**

**Table S1:** ICD-10 codes to classify diagnoses.

| Alcohol (including ethanol) | E52, F10.0–10.9, G31.2, I42.6, K29.2, K70.0–70.9, K85.2, K86.0, T51.0–51.9, Z71.4 |
| --- | --- |
| Opioids | F11.0–11.9, T40.0–40.4, T40.6 |
| Cannabinoids (including cannabis) | F12.0–12.9, T40.7 |
| Cocaine | F14.0–14.9, T40.5 |
| Nicotine | F17.0–17.9, T65.2, Z58.7, Z71.6 |
| Hallucinogens (including LSD) | F16.0–16.9, T40.8, T40.9 |
| Affective disorders | F30-39 |
| Anxiety disorders | F40-49 |
| Other mental health disorders | F00-F09, F20-29, F50-99 |
| Self-harm | X60-84 |
| Accidental poisoning | X40-49 |
| Undetermined intent | Y10-34 |
| Suicidal ideation | R45.81 |

Note: Nicotine, Cocaine, and Hallucinogens were combined with other substances due to the low frequencies.

**Figure S1**: Deaths due to suicide, accidental poisoning, and undetermined intent within 1 year following a live birth in NSW from 2002-2020.


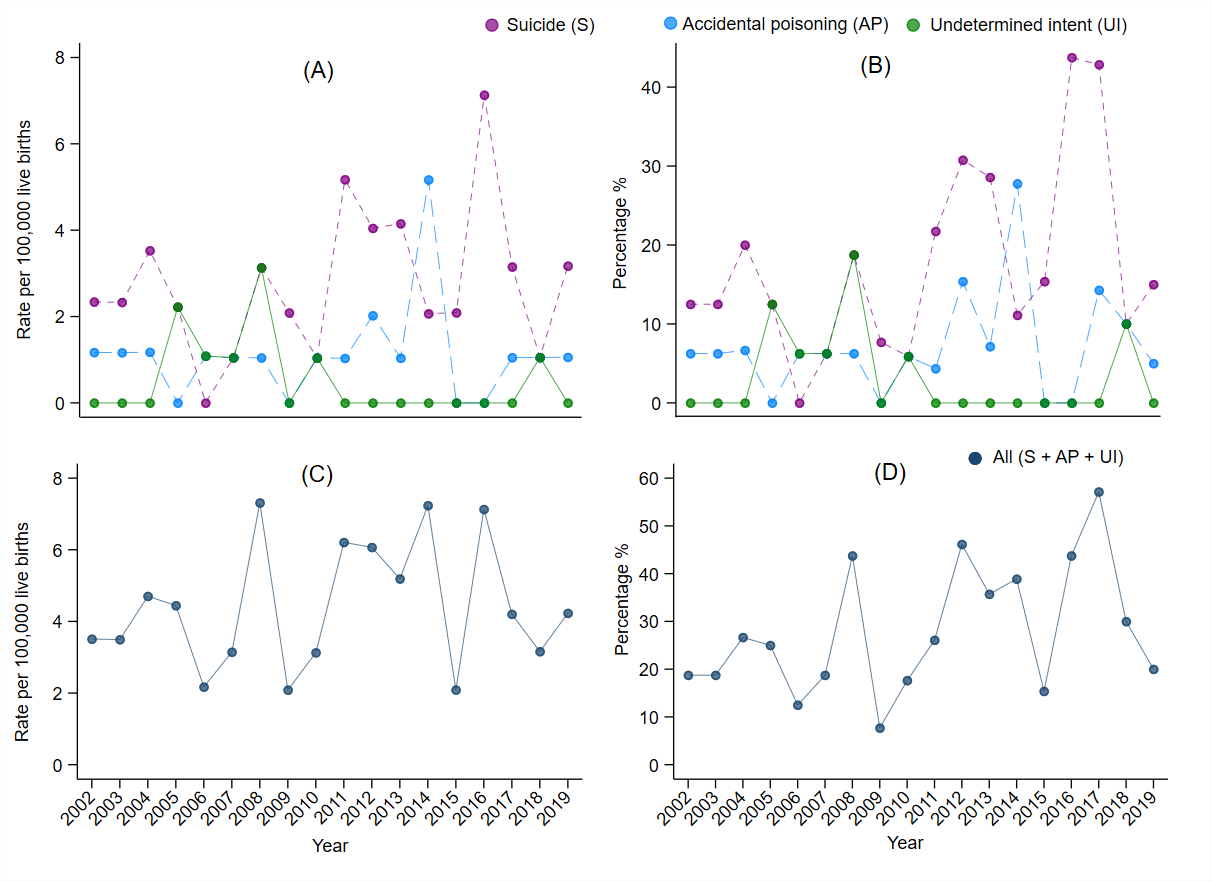
*Note: (Panel A) The rate of one-year maternal mortality by suicide, accidental poisoning, and undetermined intent per 100,000 live births; (Panel B) Maternal mortality by suicide, accidental poisoning, and undetermined intent as a percentage of all deaths within one year following a live birth; (Panel C) The rate of combined one-year maternal mortality by suicide, accidental poisoning, and undetermined intent per 100,000 live births; (Panel D) Combined maternal mortality by suicide, accidental poisoning, and undetermined intent as a percentage of all deaths within one year following a live birth. The period after 2019 was not considered due to the unavailability of a one-year follow-up period. Since a mother may give birth to multiple children (for example, twins), the rates in Panels A and C are birth-specific and capture all live births, and the rate is presented per 100,000 live births. For panels B and D, the last live birth (based on the plurality of births) was considered to calculate time to death if mothers gave multiple childbirths.*

**Figure S2**: Deaths due to suicide, accidental poisoning, and undetermined intent within 2 years following a live birth in NSW from 2002-2020.


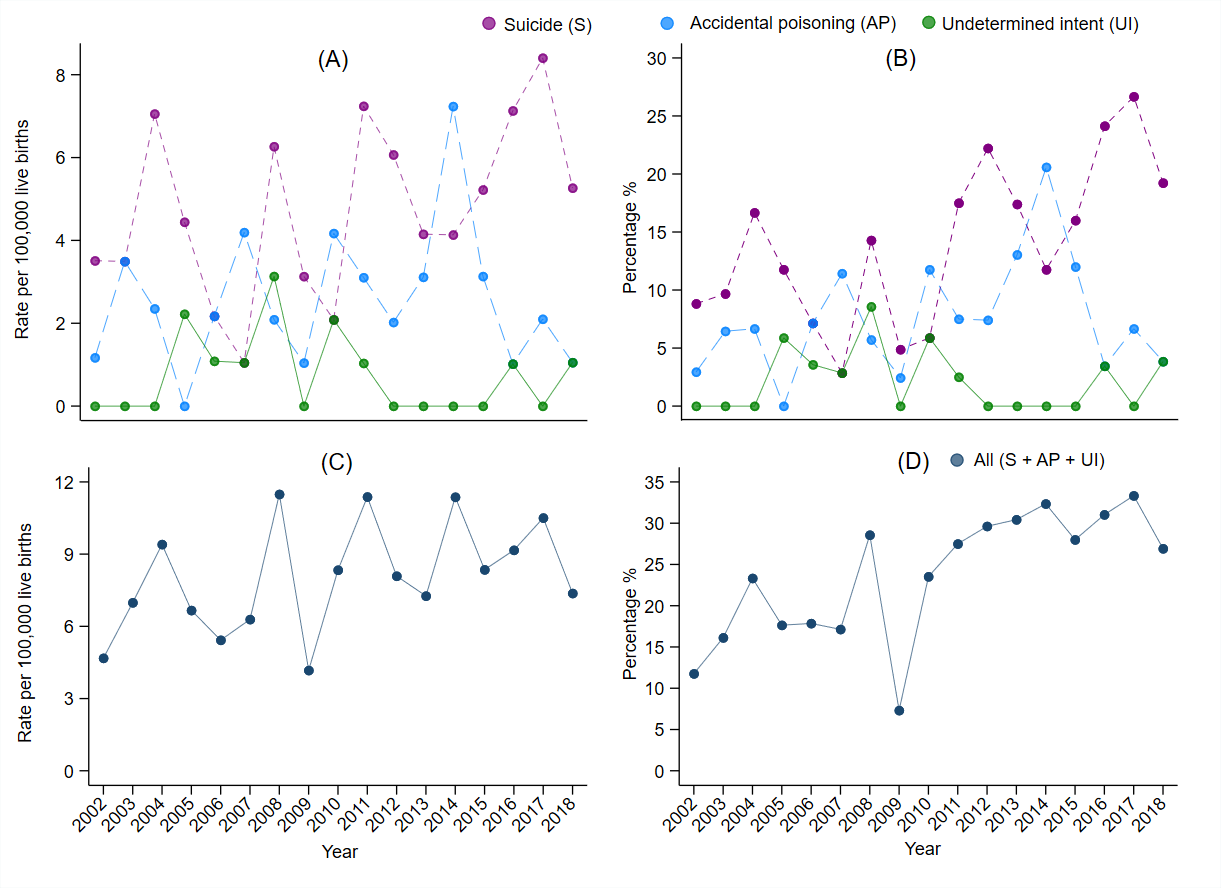
*Note: (Panel A) The rate of two-year maternal mortality by suicide, accidental poisoning, and undetermined intent per 100,000 live births; (Panel B) Maternal mortality by suicide, accidental poisoning, and undetermined intent as a percentage of all deaths within two years following a live birth; (Panel C) The rate of combined two-year maternal mortality by suicide, accidental poisoning, and undetermined intent per 100,000 live births; (Panel D) Combined maternal mortality by suicide, accidental poisoning, and undetermined intent as a percentage of all deaths within two years following a live birth. The period after 2015 was not considered due to the unavailability of a two-year follow-up period. Since a mother can give birth to multiple children at different times within two years, the rates in Panels A and C are birth-specific and capture all live births, and the rate is presented per 100,000 live births. For panels B and D, the birth date of the last live birth was considered to calculate time to death if mothers gave multiple childbirths at different time points*

**Figure S3**: Deaths due to suicide, accidental poisoning, and undetermined intent within 1-, 2-, and 5-years following childbirth in NSW from 2002-2020.


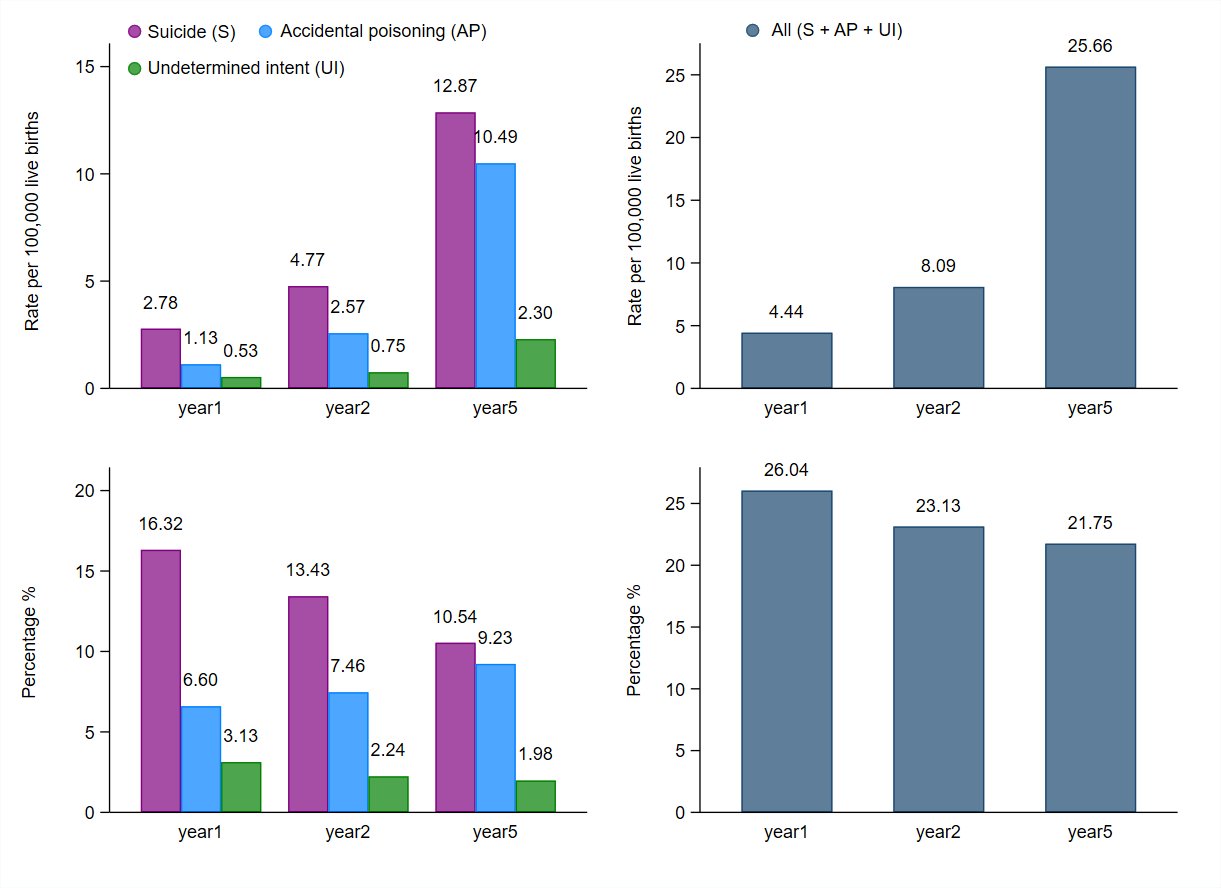


*Note: Maternal death rates per 100,000 live births and as a percentage of all deaths within 1 year, 2 years, and 5 years following childbirth are presented. When calculating the maternal death rate and percentage within 5 years, live births between 2002 and 2015 were considered. Whereas the periods from 2002-2019 and 2002-2018 were used when calculating the maternal death rate and percentage within one year and two years following childbirth, respectively. Rates are birth-specific, i.e they include multiple child births if a mother gave multiple child births at different time points, whereas the percentage calculation included unique mothers and considered the last live birth to calculate time to death.*

**Figure S4**: Timing of the maternal death following the last live birth in NSW from 2002-2020


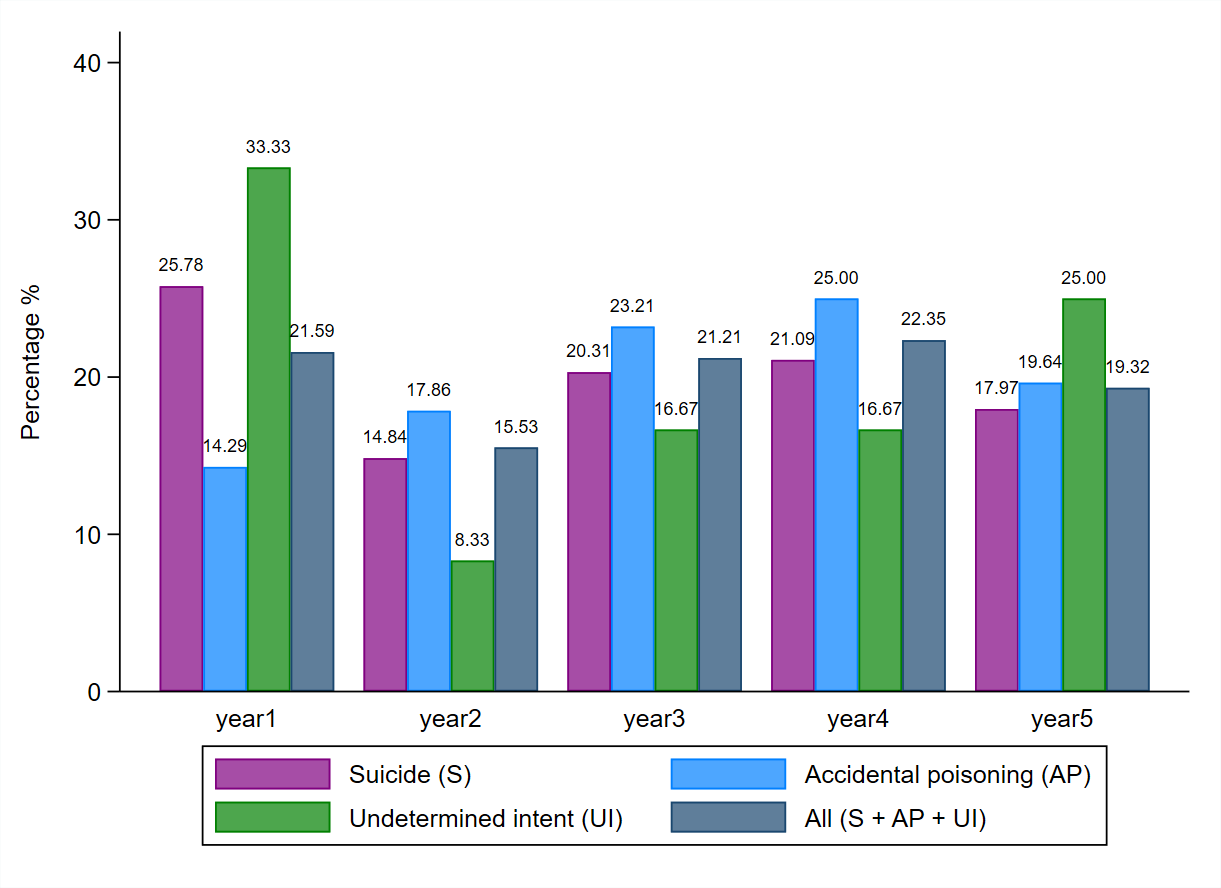


*Note: The period between 2002 and 2015 was considered with a five-year follow-up period until 2020 to calculate the timing of the death from the child's date of birth. For mothers who gave multiple births at different time points, the last live birth was considered. The values represent the percentage of deaths from a specific cause in each year, calculated out of all deaths from that cause over a five-year period (i.e., the percentages for each cause add up to 100% across five years).*

**Figure S5**: Presentations to mental health ambulatory care services among mothers who died by suicide, accidental poisoning, and undetermined intent within the last 12 months before death.


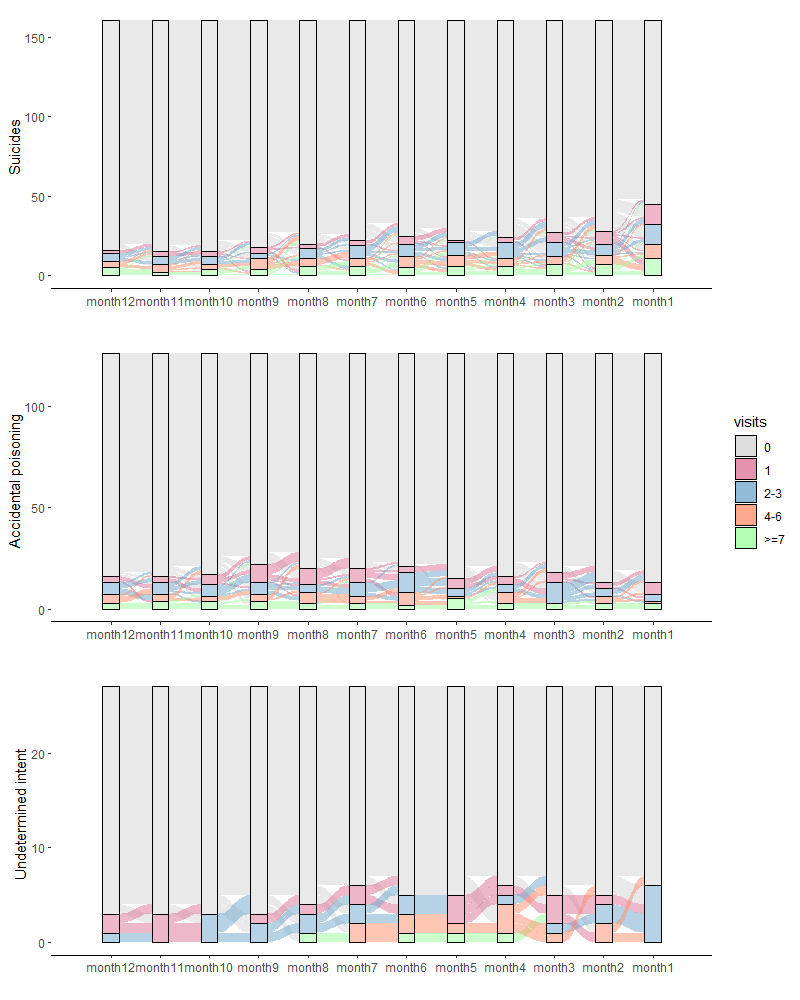


Note: *This graph shows individual mental health treatment trajectories to ambulatory care mental health services in NSW, Australia, in the 12 months before death by month. The number of mental health visits in each month was grouped into no sessions (0), 1 session, 2-3 sessions, 4-6 sessions, and >=7 sessions. The ribbons depict transitions of the number of sessions from month to month, i.e 0 sessions to 1 or +, and 1 or + to 0 sessions.*

**Table S2**: Relative risk ratios of hospital-presented conditions among mothers who died by suicide, accidental poisoning and undetermined intent compared to mothers who died by other causes and were alive for 5 years.

| Characteristics | Multinomial logistic regression | | Negative binomial models | |
| --- | --- | --- | --- | --- |
|  | baseline- alive (up to 5 years from last delivery) | baseline- deaths other causes | baseline- alive (up to 5 years from last delivery) | baseline- deaths other causes |
| Alcohol |  |  |  |  |
| Suicide | 15.78(11.13, 22.39) | 4.46(2.94, 6.75) | 15.74(11.10, 22.30) | 3.27(2.43, 4.40) |
| Accidental poisoning | 26.65(18.60, 38.20) | 7.53(4.92, 11.50) | 26.57(18.55, 38.05) | 5.12(3.75, 6.98) |
| Undetermined intent | 15.16(6.41, 35.86) | 4.28(1.76, 10.42) | 15.15(6.41, 35.83) | 4.02(1.75, 9.26) |
| Opioid/Cannabinoid |  |  |  |  |
| Suicide | 13.13(9.09, 18.96) | 2.62(1.73, 3.97) | 13.09(9.07, 18.9) | 2.21(1.60, 3.06) |
| Accidental poisoning | 69.13(48.30, 98.94) | 13.79(9.17, 20.75) | 68.75(48.07, 98.33) | 8.55(6.19, 11.81) |
| Undetermined intent | 54.99(25.74, 117.5) | 10.97(5.01, 24.03) | 54.93(25.72, 117.33) | 9.78(4.68, 20.43) |
| Mental health |  |  |  |  |
| Suicide | 9.49(6.97, 12.93) | 3.17(2.26, 4.44) | 9.48(6.96, 12.91) | 2.65(2.00, 3.52) |
| Accidental poisoning | 9.61(6.78, 13.63) | 3.21(2.20, 4.67) | 9.60(6.77, 13.61) | 2.77(2.00, 3.84) |
| Undetermined intent | 13.98(6.49, 30.12) | 4.66(2.14, 10.17) | 13.98(6.49, 30.11) | 4.45(2.09, 9.47) |
| Other substances |  |  |  |  |
| Suicide | 12.10(8.09, 18.09) | 2.52(1.59, 3.98) | 12.07(8.08, 18.03) | 2.14(1.50, 3.05) |
| Accidental poisoning | 41.30(29.00, 58.80) | 8.60(5.68, 13.03) | 41.10(28.90, 58.46) | 5.69(4.19, 7.73) |
| Undetermined intent | 44.05(20.61, 94.12) | 9.18(4.16, 20.22) | 44.00(20.60, 93.98) | 8.19(3.94, 17.00) |
| Self-harm |  |  |  |  |
| Suicide | 19.63(13.76, 28.00) | 7.07(4.49, 11.14) | 19.56(13.73, 27.87) | 4.34(3.27, 5.76) |
| Accidental poisoning | 22.10(14.96, 32.65) | 7.96(4.92, 12.89) | 22.03(14.93, 32.52) | 5.10(3.69, 7.03) |
| Undetermined intent | 33.80(15.47, 73.83) | 12.18(5.30, 27.95) | 33.77(15.47, 73.73) | 10.31(4.94, 21.55) |
| Accidental poisoning |  |  |  |  |
| Suicide | 13.61(7.72, 24.02) | 3.58(1.80, 7.12) | 13.57(7.70, 23.90) | 2.72(1.70, 4.35) |
| Accidental poisoning | 30.99(19.38, 49.57) | 8.15(4.43, 14.98) | 30.84(19.32, 49.22) | 4.95(3.43, 7.15) |
| Undetermined intent | 26.95(9.32, 77.97) | 7.08(2.29, 21.95) | 26.93(9.32, 77.82) | 6.27(2.31, 17.02) |
| Undetermined intent |  |  |  |  |
| Suicide | 12.33(5.45, 27.90) | 3.19(1.20, 8.52) | 12.30(5.45, 27.76) | 2.50(1.27, 4.93) |
| Accidental poisoning | 33.53(18.47, 60.88) | 8.68(3.87, 19.47) | 33.33(18.42, 60.32) | 4.99(3.20, 7.78) |
| Undetermined intent | 39.82(11.98, 132.33) | 10.31(2.76, 38.55) | 39.76(11.98, 131.92) | 8.56(2.87, 25.58) |
| Suicidal ideation |  |  |  |  |
| Suicide | 19.98(12.94, 30.85) | 7.14(3.98, 12.78) | 19.90(12.90, 30.67) | 4.19(3.02, 5.82) |
| Accidental poisoning | 13.12(7.39, 23.31) | 4.69(2.34, 9.37) | 13.09(7.38, 23.23) | 3.46(2.14, 5.57) |
| Undetermined intent | 4.39(0.60, 32.33) | 1.57(0.20, 11.99) | 4.39(0.60, 32.31) | 1.55(0.22, 10.99) |

*Note: Univariate analyses were performed. Confidence intervals were presented within brackets.*
